# Hill County Medical Association, Etc.

**Published:** 1891-01

**Authors:** 


ABBOTT, TEXAS, Dec. 10, 1890.

*To the Hill County Medical and Surgical Association, now in Session:*

We, your Board of Censors, have carefully examined the charges against Dr. F. B. Wilkes, and recommend his expulsion from this Association.

M. D. KNOX,
W. M. DRAKE.
B. H. VAUGHAN.

The ballot was then spread and Dr. F. B. Wilkes, of Abbott, Texas, was unanimously expelled from the Hill County Medical and Surgical Association.

---

**The Northwest Texas Medical Association** will convene in Abilene on first Tuesday in February. The association is in a flourishing condition, and we have great expectations.

The following papers will be read and discussed:

Puerperal Eclampsia. Dr. W. M. Powell.

Differential Diagnosis Diphtheria and Membranous Croup. Dr. J. M. Isbell.

Fractured Thigh. Dr. R. G. Powell.

DR. S. H. STOUT, President.

P. C. COLEMAN, Secretary.

---

**Vomiting of Pregnancy**:—Prof. Goodell, of Philadelphia, recommends the following:

| | | |
|---|---|---|
| ℞ | Ceri Oxalat | gr. i |
| | Ipecac | gr. i |
| | Creasoti | gt. ij |

M. Sig.: This to be taken every hour until nauseu is controlled.—*American Practitioner News.*

